# Strontium-90 brachytherapy following intralesional triamcinolone and 5-fluorouracil injections for keloid treatment: A randomized controlled trial

**DOI:** 10.1371/journal.pone.0248799

**Published:** 2021-03-23

**Authors:** Ke Deng, Haitao Xiao, Xiaoxue Liu, Rei Ogawa, Xuewen Xu, Yong Liu

**Affiliations:** 1 Department of Plastic and Burn Surgery, West China Hospital, Sichuan University, Chengdu, Sichuan, PR China; 2 Department of Plastic, Reconstructive and Aesthetic Surgery, Nippon Medical School, Tokyo, Japan; University of Texas Medical Branch at Galveston, UNITED STATES

## Abstract

**Background:**

Keloid disease is hard to fully eradicate. Recurrence and other unsatisfactory results were found in many patients. No current therapeutic modality has been determined to be most effective for treating keloid scars. Intralesional corticosteroid injections is most commonly recommended for primary management of small and young keloids as well as hypertrophic scars. However, it’s difficult for patients to adhere to long-term triamcinolone acetonide injection therapy because of the pain, inconvenience or complications including hormonal imbalance or irregular menstruation.

**Objective:**

We aimed to determine whether and how Strontium-90 brachytherapy as an adjuvant radiation could affect keloid recurrence after intralesional triamcinolone and 5-fluorouracil injections.

**Methods:**

We included keloid patients from March 2019 to September 2019 and randomly allocated them to two groups after 3 intralesional triamcinolone and 5-fluorouracil injections at 3 weeks interval. The experimental group received Strontium-90 brachytherapy at a total dose of 15-20Gy, while the control group didn’t receive any adjuvant treatment. We performed both Vancouver Scar Scale scoring and Color Doppler ultrasound examination to monitor and evaluate lesions regularly. A one-year follow-up was completed for each patient.

**Results:**

31 patients who had 42 keloids in total were recruited. We found intralesional triamcinolone and 5-fluorouracil injections could effectively reduce the thickness and modify the hardness of small and young keloids. Strontium-90 brachytherapy reduced the one-year recurrence rate from 85.7 percent to 44.4 percent after 3 intralesional triamcinolone and 5-fluorouracil injections. The lesions’ thickness or elasticity was not affected by Strontium-90 brachytherapy.

**Conclusion:**

Strontium-90 brachytherapy as an adjuvant radiation could effectively reduce small sized keloids recurrence after intralesional triamcinolone and 5-fluorouracil injections. It worked by enhancing the lesions’ stability post-injection.

**Trial registration:**

The clinical trial registration number: ChiCTR2000030141.

Name of trial registry: Chinese Clinical Trial Registry (http://www.chictr.org.cn/)

## Introduction

A keloid is an abnormal proliferation of scar tissue that forms at the site of cutaneous injury. It grows beyond the original margins of the scar and rarely regresses [1]. Various theories have been proposed to explain the underlying etiology and pathogenesis of keloid formation [2, 3]. Strategies including inflammation modification, collagen metabolism regulation and surgical manipulation of the keloid scar have been suggested for keloid therapy [4, 5]. Surgical excision, intralesional injection of steroids, verapamil and 5-fluorouracil (5-FU) usage, cryotherapy, laser therapy, radiation therapy and combination therapy are effective therapeutic approaches recommended for patients under different conditions [1]. However, no single therapeutic modality has been determined experimentally to be most effective for treating keloid scars. Full eradication is hard to be achieved. Keloid recurrence and other unsatisfactory results were found in feedbacks of many patients.

Intralesional corticosteroid injections have been used for pathologic scar treatment since the mid-1960s [6, 7]. It is most commonly recommended for primary management of small and young keloids as well as hypertrophic scars [8, 9]. It has been suggested Triamcinolone acetonide (TAC) injections combined with other treatment modalities, such as surgery, 5-Fu injection, laser therapy and cryotherapy could achieve better therapeutic results [10]. It was evidenced that injection of TAC combined with 5-Fu could better induce lesion atrophy and significant hyperemia improvement compared with injection of only 5-Fu or interchangeable injection of 5-Fu and TAC [11]. TAC combined with 5-Fu injection treatment has been written into the Chinese Expert Consensus on clinical prevention and treatment of keloids [12]. Multiple and repeated injections and long-term treatment courses were advocated to achieve persistent inhibition of scar hyperplasia [13–16]. However, some patients would give up during treatment course because of hormonal imbalance or irregular menstruation [13], others found it was difficult to adhere to long-term TAC injection therapy because of the pain or the inconvenience caused by repeated hospital visits. As a result, keloid recurrence could be easily found in patients failed to adhere to long-term injection treatment.

Superficial X-rays, electron beams and low- or high-dose-rate brachytherapy have been employed primarily as an adjunct to surgical removal of keloids, with overall good results in terms of reduced recurrence, implying that radiotherapy has an superior effect in preventing the recurrence of keloids [17, 18]. Radioisotopes therapy is a type of brachytherapy that delivers radiation within the immediate target area using a small delivery apparatus attached to the external surface of the skin. It ensures more focused *in situ* delivery and distribution of radiation to the target area [19]. Beta-rays released from Phosphorus-32 and Strontium-90 (90Sr) and gamma-rays released from Cobalt-60, Iridium-192 are the commonly used radioisotopes radiation sources [20, 21]. In this study, we investigated whether and how 90Sr brachytherapy could reduce keloid recurrence rate after intralesional TAC+5-Fu injections, and suggest a possible minor invasive treatment strategy for small and young keloid scars.

## Methods

### Ethical considerations

Before conducting the study, the project has been reviewed and approved by the Bioethics Committee of West China Hospital of Sichuan University (approval 2019 (25)). The trial was retrospectively registered with the Chinese Clinical Trial Registry (ChiCTR2000030141). We humbly confess the delay in registering the clinical trial that happened partly due to the lack of knowledge about trial registration. When we considered ethic and legal issues, we came to the conclusion that this registration was not mandatory. We confirm that all ongoing and related trials for this intervention are registered. Informed consent forms were used to inform all eligible patients about the purpose and plan of the study and how data of the study would be used and stored. Written forms of consent were obtained from all participants.

#### Inclusion criteria

(1) Patients met the clinical diagnostic criteria of keloids: scar-like structures resulted from wound healing, protruding skin surface and infiltrating beyond original damage area. It might be accompanied by itching, pain and other discomfort symptoms. (2) The disease course was longer than 2 years and no spontaneously shrinking tendency was noticed. (3) Patients signed the informed consent and agreed to complete the follow-up procedure including VSS (Vancouver Scar Scale) assessment and color Doppler ultrasound assessment at the designated visit time.

#### Exclusion criteria

(1) Patients who were previously treated with surgery, radiation, injection, laser, cryotherapy, etc. within 3 months. (2) Patients refused to accept ingredient including TAC or 5-Fu; (3) Patients with keloids that experienced infection or ulceration. (4) Patients with other skin conditions such as rash; (5) Patients with systemic diseases such as hypertension, diabetes, gout, or immune diseases; (6) Patients allergic to TAC or 5-Fu; (7) Patients with ear lobe keloids; (8) Patients with keloids larger than 9 cm^2^ or thicker than 4mm.

### Procedure

We assessed all keloid patients for eligibility at the clinic of our hospital. Patients eligible for inclusion were asked for written informed consent prior to the participation in this study. For recruited patients with multiple lesions, the dominant one lesion or two lesions on different body regions (praecordium, scapula, abdomen, pubis or limbs) were defined as the experimental objects. TAC+5-Fu injections were given to all included patients. Then, patients were randomly allocated to experimental and control groups after injection treatment was completed. The experimental group received 90Sr brachytherapy at a total dose of 15-20Gy, while the control group didn’t receive any adjuvant treatment. We recorded the age, sex, keloid site, treatment, and subjective symptoms of all included patients immediately after the enrollment.

### TAC+5-Fu injections method

0.6 ml 2.5% 5-FU was added to 5 ml 1% TAC and then mixed with 1 ml 2% lidocaine. The total mixture, a dose of 0.2 ml/cm^3^, was injected intralesionally. Three times of injection were performed for each keloid with an interval of 3 weeks.

### Brachytherapy method

90Sr brachytherapy was only given to the experimental group 3 weeks after the final injection. A 28mm × 28mm 90Sr skin applicator with 20mm × 20mm effective field was applied to cover each keloid lesion area (1.81mGy/s). Seventy-five percent of β-rays emitted by 90Sr are absorbed by the first 2mm of tissue and much of the remainder by the next 1mm. The surrounding normal skin was protected by lead. A total dose of 15-20Gy was given continuously for 3 or 4 days depending on the involved body area. Keloids on the chest wall, scapular or suprapubic region were treated by a total of 20Gy radiation in 4 fractions over 4 days. Keloids on other regions were treated by a total of 15Gy radiation in 3 fractions over 3 days. The difference in dose according to the body region treated was decided based on recommendation of site-specific radiation protocols post-surgery considering the recurrence rate of resected keloids on the chest wall, scapular or suprapubic region are comparatively high [22, 23]. The treatment would be stopped if any dermatitis, skin swelling, infection or ulceration appeared.

### Follow-up strategy

Both groups were followed up regularly for at least one year after the treatment. VSS scoring was performed before the patients’ injection, 3 weeks after the last injection and monthly after the brachytherapy completion or any time patients visited for recurrence concerns. Color Doppler ultrasound examination was performed before the patients’ injection, 3 weeks after the last injection, 6 months after the brachytherapy completion and when a relapsed case was screened out by VSS assessment. If a patient’s VSS thickness score increased by 1 after the whole treatment course was completed, he or she would be sent for a color Doppler ultrasound examination to confirm the recurrence. When a lesion’s thickness increased by 2mm assessed by ultrasound, we considered it as a recurrent case. In this case, the follow-up would be ended and an extra injection would be performed to prevent the lesion from getting worse. Complications such as hyperpigmentation, menstrual disorders, local depression, skin ulceration, and Cushing syndrome were recorded during the follow-up.

We used Philips IU22 ultrasonic diagnostic instrument to assess dermal thickness and pliability. Muscle and bone examination mode was set and a 4-15mhz linear array probe was used to measure the thickness of keloids. The thickest parts of the lesions were measured 3 times and the average values were taken as the lesions’ thickness in mm. Then, the machine was switched to shear wave elastography (SWE) mode. The default range of Young’s modulus was set as 0–600 kPa. We placed the 4-15mhz linear array probe vertically on the skin surface and the Q-box was placed in the area with the maximum velocity value. We captured an region of interest (ROI), a round sampling frame measured 7–10 mm in diameter within the Q-box (S1 Fig). After the color in ROI was completely filled and stabilized for 3s, the sampling frame was fixed and the Young’s modulus were measured. The Young’s modulus were measured for 3 times for each keloid and the average value was taken as the keloid’s elastic modulus.

### Sample size

We used G*Power software (version 3.1.9.3) to calculate the required sample size. The effect size of the primary outcome (the mean ultrasonic thickness of keloids examined at the 6-month post-brachytherapy visit or any relapse-identified visit before that) was expected based on our preliminary experimental data. Based on an estimated effect size of d = .83, alpha level = .05 (normal level of significance, one-tailed as direction was hypothesised), power = .80 and unpaired t test, a total sample size of n = 38 lesions was generated. Therefore, we aimed to recruit 21 lesions per group based on a 10% dropout rate.

### Randomisation and blinding

The randomization sequence was computer generated by an experimenter who was not involved in recruitment, assignment of participants to groups, or conducting assessments. The randomization schedule was managed by the research assistant responsible for recruitment and enrolling eligible participants. The VSS assessments were independently performed by two investigators who were masked to patients’ allocation information. If the scores of the two were different, the investigators would discuss to reach an agreement. The ultrasound assessments were performed by an investigator who was masked to any information relating to the allocation and the VSS data. At the conclusion of the baseline assessment, the participants were informed of their group allocation. In order to reduce the risk of bias, subjective outcomes such as patients’ symptoms were avoided and the outcome measures were investigator-reported or objective (e.g. color Doppler ultrasound examination), therefore these variables should not have been influenced by participants knowing group allocation.

### Data analysis

Quantitative data was expressed as mean ± sd. Effect size and 95% CI was displayed where appropriate. Categorical data was described by frequency and percentage. The intra-group mean difference of quantitative data was analyzed by paired t-tests, and the difference between groups was analyzed by two independent sample t-tests. For the relapse analysis, we conducted a log-rank test to compare the relapse distribution of two groups. All data was analyzed and charts were generated by using GraphPad Prism 7.0 software. The significance level was defined as 0.05.

## Results

**Fig 1:**
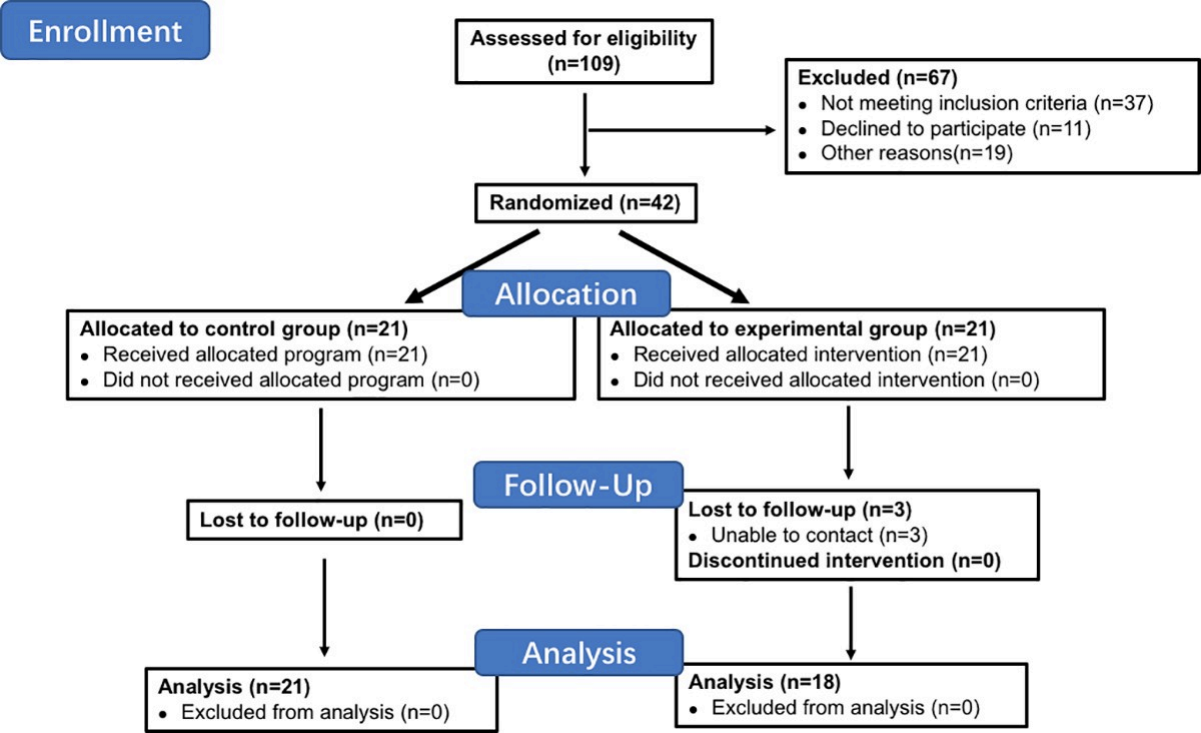
Flow chart of this study.

**Fig 2:**
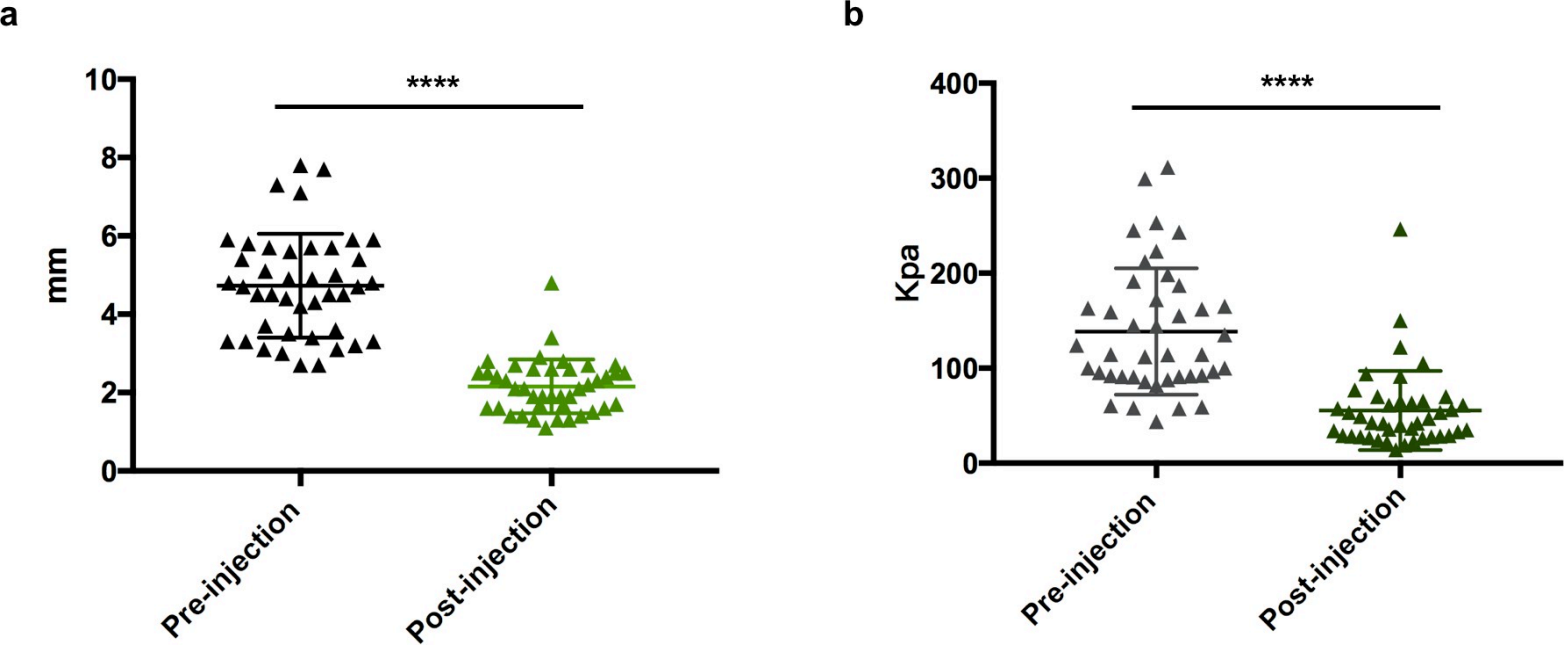
The effect of TAC+5-Fu injection therapy. TAC+5-Fu injection reduced (a) thickness and (b) elastic modulus of keloids measured by Doppler ultrasound. ****p < 0.0001.

We assessed 68 patients with 109 lesions for eligibility and enrolled 31 patients consisting of 12 males and 19 females from March 2019 to September 2019 (Fig 1). The 31 recruited patients who had 42 lesions altogether received TAC+5-Fu injections. We performed VSS scoring and color Doppler ultrasound examination right before and 3 weeks after the injection was completed. The results showed that TAC+5-Fu injection could effectively reduce the thickness and modify the hardness of keloids as indicated by ultrasonic imaging of soft tissue thickness and elastic modulus. TAC+5-Fu injection significantly thinned lesions by an average of 2.57mm (4.73 ± 0.20 vs. 2.16 ± 0.11 mm immediately before and 3 weeks after injection, p<0.0001) (Fig 2A). Elastic modulus were reduced by an average of 83.14Kpa post-injection (138.60 ± 10.28 vs. 55.47 ± 6.45 Kpa immediately before and 3 weeks after injection, p<0.0001) (Fig 2B). We did not observe any relapsed cases before allocation.

**Fig 3:**
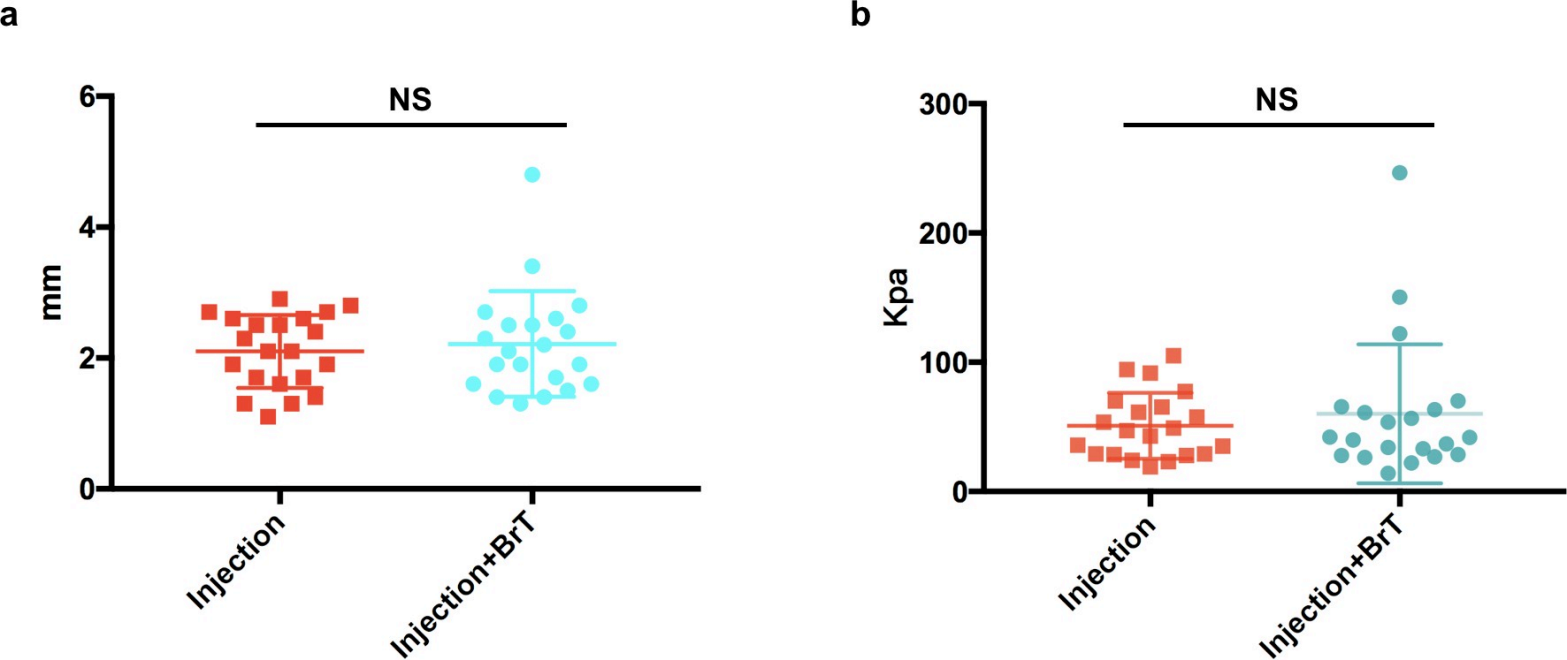
Baseline difference testing between the experimental group and the control group. There was no significant difference between two groups in terms of (a) lesions’ thickness or (b) ultrasonic elasticity. NS, p > 0.05.

After TAC+5-Fu injection was completed for each patient, 15 patients who had 21 keloids in total were randomly allocated to the experimental group and 16 patients who had 21 keloids in total were allocated to the control group. The median age of patients in both groups were 24 years old. There were 6 male patients in both groups and one more female patient in the control group than experimental group (10 vs 9). The lesions’ sites included praecordium, scapula, abdomen, pubis and limbs. Among them, praecordium is the most common spot with 15 out of 21 (71.4%) lesions in the experimental group and 14 out of 21 (66.7%) lesions in the control group (Table 1). In order to prove that the lesions of the two groups were comparable before 90Sr brachytherapy was performed, we compared their ultrasonic thickness and elasticity respectively after injection treatment. It was confirmed that there was no statistically significant difference in thickness or elasticity between the two groups (p = 0.596, p = 0.478) (Fig 3A and 3B).

**Table 1:** Demographic information of recruited patients and keloids.

	Experimental group	Control group
**Age (years): median**	24 (19–41)	24 (21–48)
**Gender**	15	16
Female	9 (60.0%)	10 (62.5%)
Male	6 (40.0%)	6 (37.5%)
**Sites**	21	21
Praecordium	15 (71.4%)	14 (66.7%)
Scapula	3 (14.3%)	4 (19.0%)
Abdomen or pubis	1 (4.8%)	2 (9.5%)
Limbs	2 (9.5%)	1 (4.8%)

**Fig 4:**
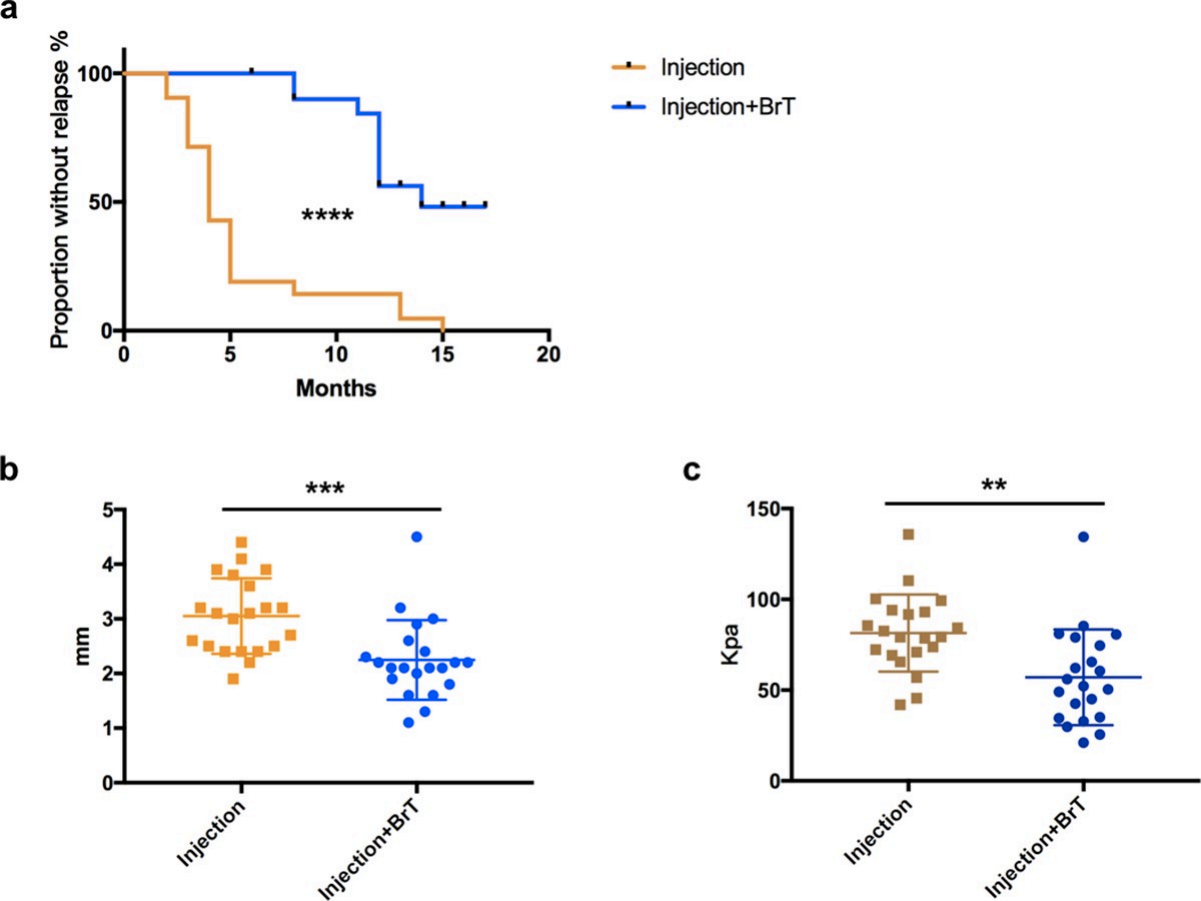
90Sr prevent keloid from recurrence after injection treatment. (a) relapse analysis of experimental and control groups. (b) ultrasonic tissue thickness comparison between the two groups measured at 6-month post-brachytherapy or before (relapsed). (c) ultrasonic elastic modulus comparison between the two groups measured at 6-month post-brachytherapy or before (relapsed). **p < 0.01; ***p < 0.001; ****p < 0.0001.

We successfully followed up with 92.9% (39 out of 42) cases following the strategy described above. However, we were not able to contact 2 patients who had 3 lesions in total in the experimental group after the 6- and 8-month post-brachytherapy visits respectively. A total of 26 lesions experienced relapse within the one-year follow-up period (Fig 4A). The relapse curve indicated that 90Sr brachytherapy could significantly reduce keloid recurrence and keep the lesions in an inactive state after the treatment course had ended (p<0.0001) (Fig 4A). During the one-year follow-up after brachytherapy was completed, we identified recurrence for 8 out of 18 (44.4%) lesions in the experimental group and 18 out of 21 (85.7%) in the control group (Fig 4A). The median relapse-free time of the control group was only 4 months. We continued following up with the 13 unrelapsed cases until we collected all data in October 2020. Nine of the cases that received brachytherapy remained stable under our observation with median follow-up time of 14(12–17) months. The only 3 cases in the control group that did not relapse within the one-year follow-up experienced recurrence at 13, 13 and 15 months respectively.

To quantify the difference that 90Sr brachytherapy made to keloids that received injection therapy, we compared the tissues’ thickness and ultrasonic elasticity examined at the 6-month post-brachytherapy visit or any relapse-identified visit before that. We found both the tissue thickness and ultrasonic elasticity of lesions that received 90Sr brachytherapy post-injection were significantly lower than those that only received injections (p<0.001, p<0.01) (Fig 4B and 4C). 81 percent (17 out of 21) of keloids in the control group experienced recurrence within 6 months after injection. Their mean thickness was 3.05± 0.15mm, while the mean thickness of the experimental group was 2.25 ± 0.16mm. The mean elastic modulus of keloids in the control group was 42.8 percent higher than those in the experimental group (81.43 vs. 51.03 Kpa).

**Fig 5:**
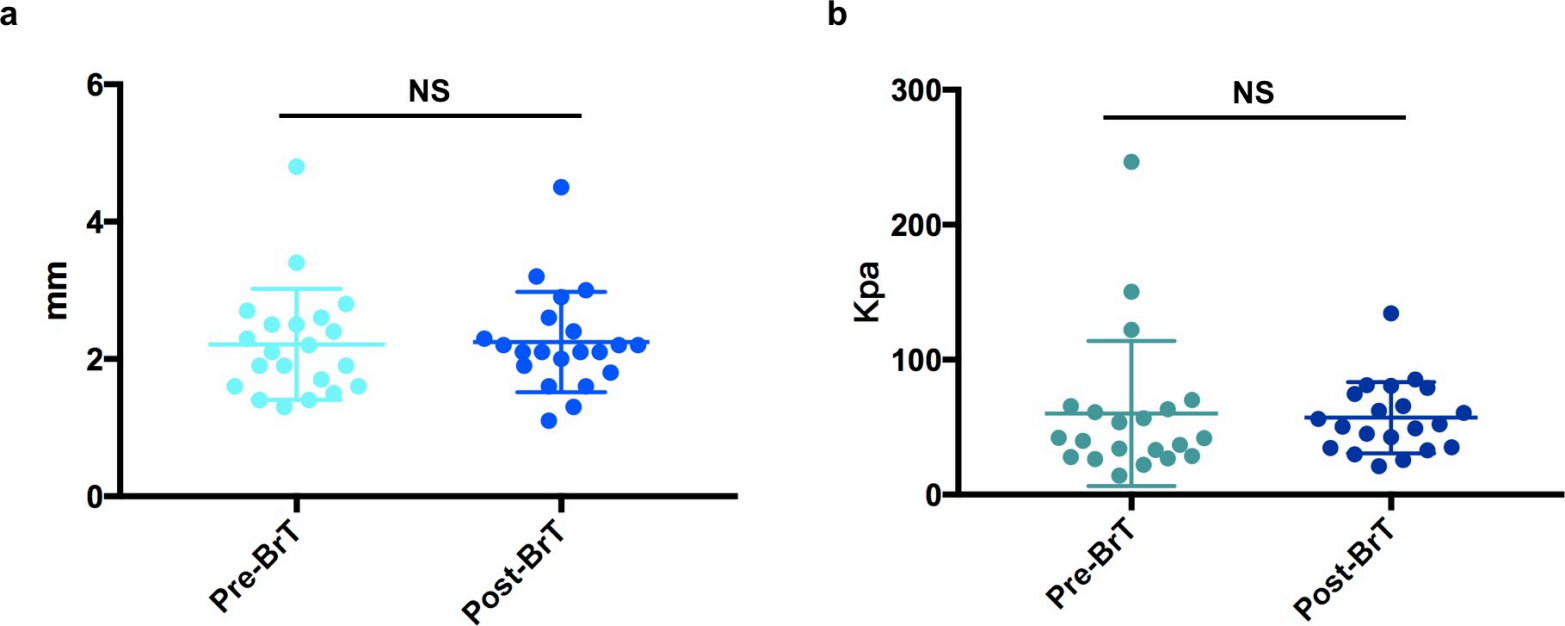
The effect of 90Sr brachytherapy. 90Sr brachytherapy did not affect (a) the lesions’ thickness or (b) elastic modulus post-injection. NS, p > 0.05.

In order to figure out how 90Sr brachytherapy could affect keloids after injection treatment, we analyzed the keloids’ thickness and elastic modulus right before and 6 months after brachytherapy. Interestingly, no significant difference was detected in both lesion thickness and elasticity (p = 0.788, p = 0.706) (Fig 5A and 5B). Besides, we did not observe any significant skin depression or ulceration after 90Sr brachytherapy was conducted. It implied that 90Sr brachytherapy could help lesions remain stable after keloids were thinned by TAC+5-Fu injection therapy.

**Fig 6:**
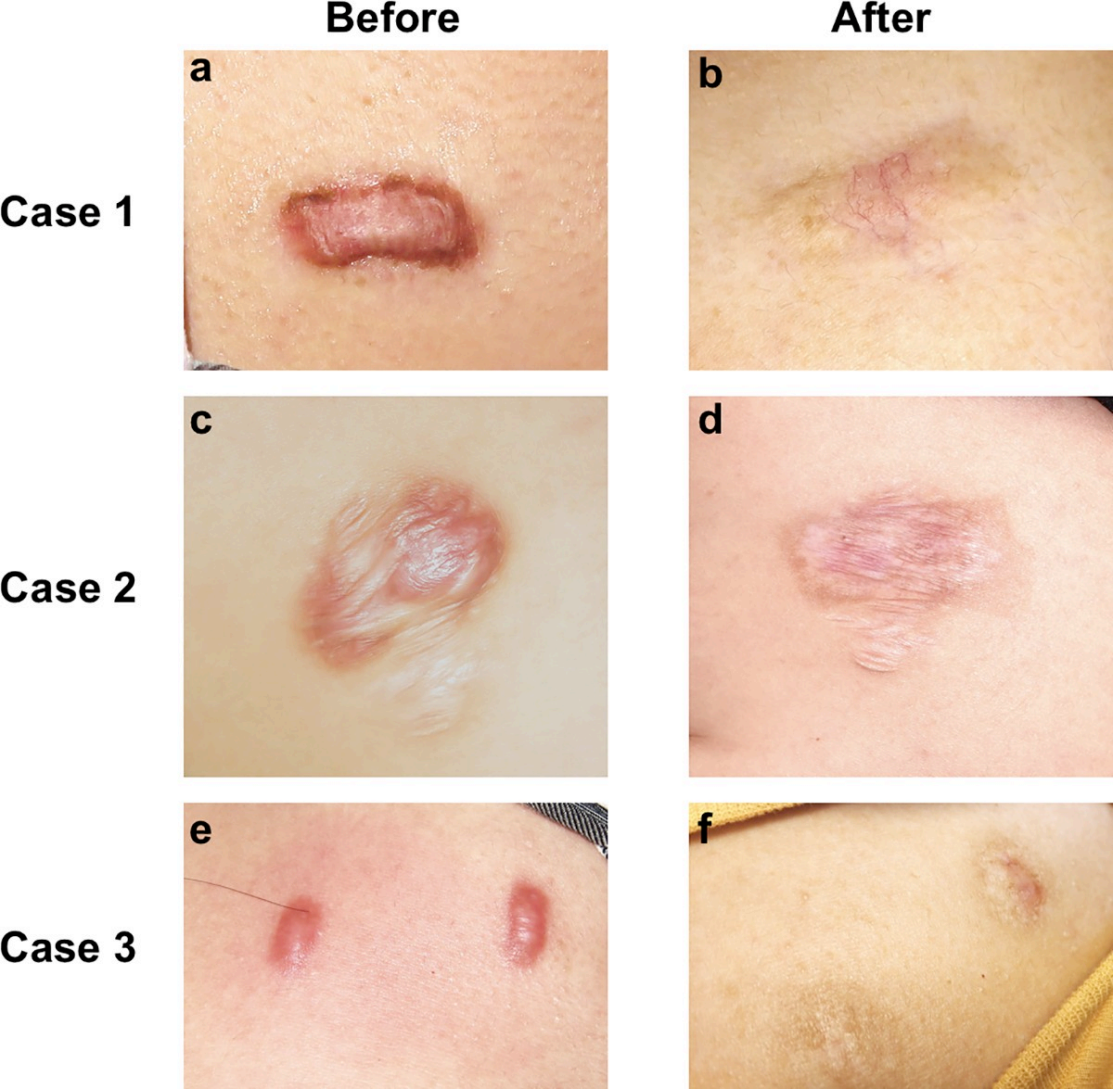
Case 1. A praecordial keloid scar in a 25-year-old women before treatment and 16 months after TAC+5-Fu injection followed by 90Sr brachytherapy. **Case 2.** A scapular keloid scar in a 23-year-old women before treatment and 14 months after TAC+5-Fu injection followed by 90Sr brachytherapy. **Case 3.** A praecordial keloid scar in a 22-year-old women before treatment and 17 months after TAC+5-Fu injection followed by 90Sr brachytherapy. Scale bar, 2cm.

We present 3 representative cases that did not show any sign of recurrence 16 months, 14 months and 17 months post-brachytherapy (Fig 6). For most patients, flattening, normalization of pigmentation, vascularity and pliability of lesions were well obtained by TAC+5-Fu injection. The recurrence reduction and long-term disease control could be achieved by an adjuvant 90Sr brachytherapy.

## Discussion

In this study, we assessed the effectiveness of 90Sr brachytherapy after TAC+5-Fu injection therapy for keloid management by conducting a perspective randomized controlled trial. In addition, we assessed the effectiveness objectively and quantitatively by using ultrasonic imaging of soft tissue thickness and elasticity. We found 90Sr brachytherapy as an adjuvant radiation could effectively reduce keloid relapse after a nine-week course of 3 TAC+5-Fu injections. It enhanced the lesions’ stability post-injection and prevented the lesions from re-growth.

The adequate application of injection therapy before Sr90 radiation is the key procedure to achieve success of treatment. Although dermis varies between 1–4 mm on different locations, its structure and thickness would be reconstructed by keloid when the lesion raised. In order to bring entire lesions into the 2-3mm’ effective range of 90Sr isotope ray, we innovatively induced lesions’ atrophy and flattening by a treatment course of 3 TAC+5-Fu injections. We confirmed the lesions’ thickness (2.16 ± 0.11 mm) before Sr90 was applied to ensure the entire lesion to be fully covered by the effective range (2-3mm) of beta rays released from 90Sr and prevent off-target radiation and subsequent complications. In this way, the focused delivery of radiation, concentrated energy and precision would be ensured and the subsequent keloid recurrence inhibition could be achieved. Fibroblasts proliferation suppression and the synergistic effects with TAC in terms of inhibiting wound healing inflammation, enhancing collagen and fibroblast degeneration could be possible mechanisms by which 90Sr isotope ray worked [4, 19]. The underlying mechanisms of how 90Sr inhibited keloid recurrence are to be elucidated by more fundamental research.

The recurrence rate of our control group was 85.7%, far exceeding the recurrence rate of TAC+5-Fu treatment reported in previous literatures [14]. This result could be attributed to the following reasons. First of all, keloid relapse was recognized as scar re-proliferation and beyond the original incision in most publications. In this study, we considered lesion’s VSS thickness score increased by 1 and ultrasound thickness increased by 2mm as recurrence after treatment, which is much more aggressive than the recommended relapse criteria. We believe it was sensible to both recognize the lesions’ hyperplasia and intervene the scar tissue regrowth early instead of waiting until diagnostic criteria of recurrence was met. In this way, no surgical excision would be needed to treat small sized lesions. The expansion of recurrence diagnostic indications could very possibly be beneficial for the long-term disease control, but at the same time, resulted in a much higher recurrence rate. Second, we excluded patients with earlobe keloids in this study because surgical excision and immediate postoperative radiation has been suggested as a preferred therapy for earlobe keloids. It could lead to a recurrence rate under 10% and prevent side effects which include hypo- or hyperpigmentation, telangiectasia, skin atrophy and pain upon injection [22, 24, 25]. The recurrence rate of earlobe keloids was significantly lower than keloid on other sites, especially on anterior chest wall regions [22]. Therefore, the recurrence rate of our research was relatively higher than others. Finally, in order to shorten the treatment course and reduce complications associated with long-term TAC treatment, we only performed 3 injections for each patient. It was not surprising to find that the recurrence rate was higher compared with those in research that performed longer courses of injection treatment. In case of an unaccepted recurrence, the patient is still candidate for other treatments such as excision followed by Ir192 brachytherapy [26].

Surgery combined with radiotherapy is a recommended method most likely to cure keloid after one course of treatment. However, around 15%-30% of the keloid patients who received surgical excision followed by adjuvant irradiation would suffer from relapse depending on the lesions’ site [22, 27]. It remained a difficult problem for plastic surgeons and dermatologists to treat patients with recurrent refractory keloids and patients with multiple keloids who were not suitable for surgery. TAC+5-Fu injection combined with adjuvant 90Sr brachytherapy can be recommended for primary management of multiple small and young keloids. Furthermore, it could possibly be an alternative treatment for recurrent refractory keloids.

The results of this study are to be verified by clinical trials with longer follow-up and larger case volumes. We observed pigmentation in some cases which remained a cosmetic concern for some patients even though the keloid tissue was diminished. We are looking forward to studies that further explore modifications of this strategy or any other minor invasive strategies to achieve better outcomes in terms of patients’ compliance, recurrence avoidance and cosmetic outcomes for keloid patients.

In conclusion, TAC+5-Fu injection could effectively reduce the thickness and modify the hardness of small and young keloids as demonstrated by ultrasonic imaging of soft tissue thickness and elasticity. 90Sr brachytherapy could effectively prevent keloids from recurrence after 3 TAC+Fu-5 intralesional injections. It worked by enhancing the lesions’ stability post-injection, but not affecting the thickness or the elasticity of the tissue. As a result, the skin integrity was well preserved and no ulceration was observed during the treatment course. The whole treatment course of this minor invasive treatment strategy only included 3 TAC+5-Fu injections at 3 weeks interval and 3 or 4 days of brachytherapy. This practice, to a great extent, has decreased the pain, the inconvenience and the complications associated with long-term and repeated TAC injections and improved patients’ compliance. It was suggested that Strontium-90 brachytherapy following 3 TAC+Fu-5 injections could be a promising novel treatment strategy for small sized keloid scars.

## Supporting information

S1 Fig The Young’s modulus of a keloid assessed by ultrasonic Shear Wave Elastography (SWE) mode. (TIF)

S1 File Completed CONSORT checklist. (PDF)

S2 File Trial study protocol (Chinese). (PDF)

S3 File Trial study protocol (Translated). (PDF)
