# Supplementary figures and images for: Strontium-90 brachytherapy following intralesional triamcinolone and 5-fluorouracil injections for keloid treatment: A randomized controlled trial
Source: PLoS One. 2021 Mar 23;16(3):e0248799. doi: 10.1371/journal.pone.0248799 (PMC7987169; doi:10.1371/journal.pone.0248799)

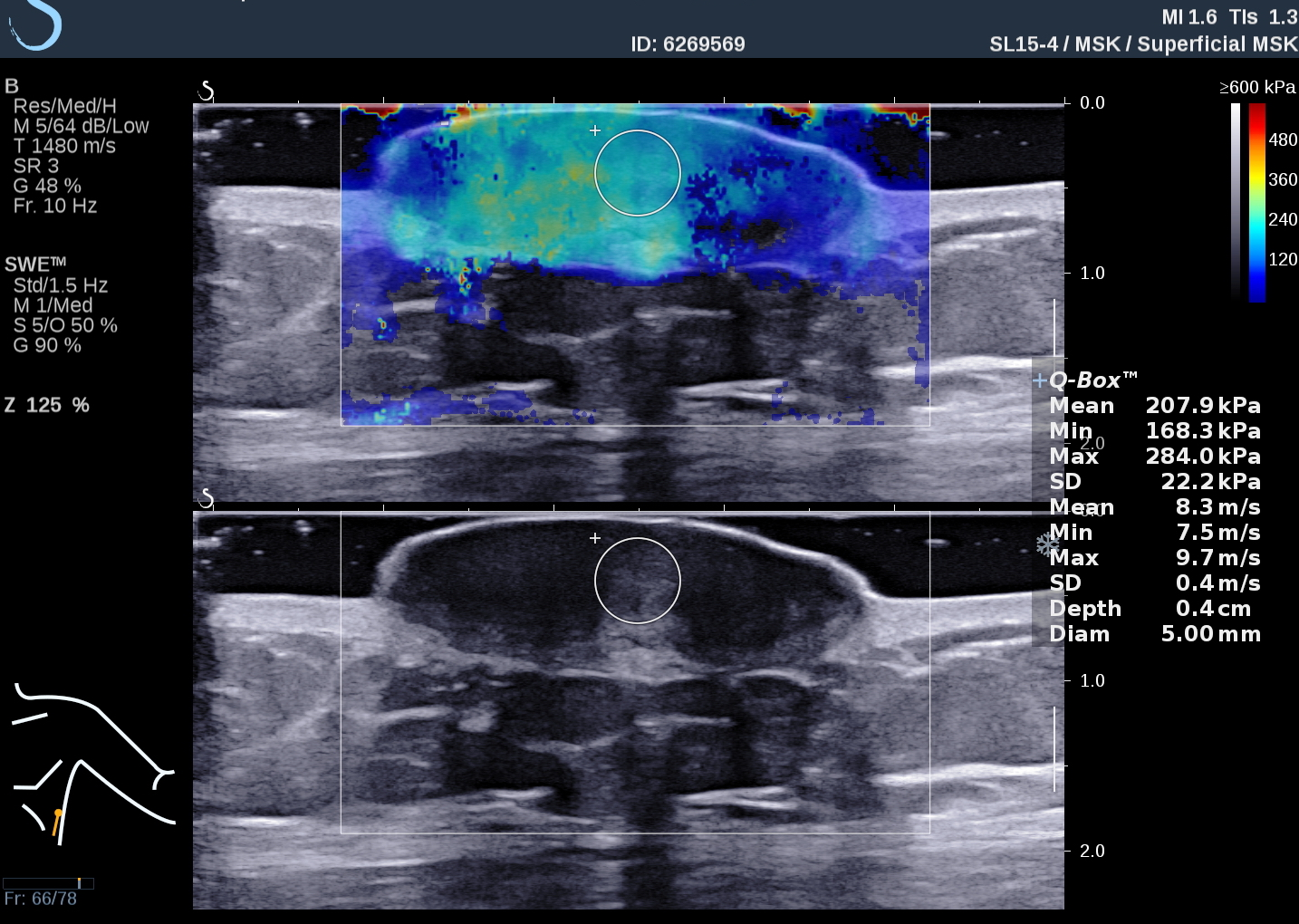

Supplement: S1 Fig — (TIF) [file pone.0248799.s001.tif]
