# Supplementary material for: Strontium-90 brachytherapy following intralesional triamcinolone and 5-fluorouracil injections for keloid treatment: A randomized controlled trial
Source: PLoS One. 2021 Mar 23;16(3):e0248799. doi: 10.1371/journal.pone.0248799 (PMC7987169; doi:10.1371/journal.pone.0248799)
Supplement: S2 File — (PDF) [file pone.0248799.s003.pdf]

生物医学伦理研究方案

（干预性临床研究）

研究题目：改良注射方法及联合 Sr90 核素照射治疗瘢痕疙瘩的临床疗效评估

研究单位：四川大学华西医院

研究科室：美容整形烧伤外科

申办者：肖海涛

研究负责人（签名

联系电话：18980606392

日　期：2019 年 1 月—2020 年 7 月

版本号：V2.1

方 案 摘 要

**研究设计**<br>**(可多选)**	**☐病例对照研究　　☐队列研究　　☐横断面研究**<br>**√随机对照研究　　☐应用盲法　　☐其他：**
**研究类型**<br>**（请根据项目类型勾选）**	**（A 类：高风险）**<br>☐ 基因编辑研究<br>☐ 细胞治疗研究<br>☐ 植入性医疗器械研究（含 3D 打印）<br>☐ III类临床新技术（安全性、有效性确切，技术难度大、风险高）<br>☐ 特殊人群研究（儿童、孕妇、智力低下者、精神障碍受试者等）<br>☐ 超药物说明书研究（☐超适应症　☐超给药途径　☐超剂量　☐超年龄　☐超禁忌症　☐超人群　☐其他，请说明：　　）<br>☐ 超器械说明书研究（☐超适应症　☐使用范围　☐超禁忌症　☐超人群　☐其他，请说明：　　）<br>☐ 其他（研究者判定，请说明：　　）<br>**（B 类：中风险）**<br>☐ 上市后生物制剂研究（预防用和治疗用）<br>☐ 上市后治疗性疫苗研究<br>☐ 上市后罕见病药物研究<br>√ II类临床新技术（安全性、有效性确切，有一定技术难度，有一定医疗风险和伦理风险）<br>☐ 其他（研究者判定，请说明：　　）<br>**（C 类：低风险）**<br>☐ 已上市 5 年药物研究（包括化药、仿制药等）<br>☐ 已上市器械研究（含 AI，影像软件）<br>☐ I类临床新技术（安全性、有效性确切，技术难度低、几乎不存在伦理风险的医疗技术）<br>☐ 其他（研究者判定，请说明：　　）

**病例总数**	122
**风险/受益分析**	改良的注射方法可以减少患者注射时的疼痛感，加快瘢痕疙瘩萎缩的速度。但相较于传统注射方法，由于注射方法更靠近基底，可能增加组织凹陷的风险。注射后增加辅助性放疗，有望减少瘢痕疙瘩患者复发率。小剂量放疗可能增加色素沉着等风险。
**风险判断**	□不大于最小风险　　√大于最小风险<br>最小风险：指试验中预期风险的可能性和程度不大于日常生活、或进行常规体格检查或心理测试的风险
**研究期限**	2019 年 1 月至　2020 年 7 月

1 研究背景

瘢痕疙瘩是由于成纤维细胞的异常增殖和细胞外基质累积形成的一种疾病。临床表现为质硬，突出皮面的瘢痕突起，常伴有瘙痒、疼痛等不适感觉。与普通瘢痕的区别主要在于病变的大小超过原始损伤的范围，呈蟹足状向周围浸润性生长，不会自行的萎缩。

瘢痕疙瘩以往主要采用温哥华量表进行评价。温哥华量表主要是通过面诊的医生判断瘢痕疙瘩的厚度、硬度、颜色、大小，并据此加以主观的评分。但由于主观性太强，不同医生之间评价的差异性较大。后来有人逐渐引入彩超、三维 CT 等手段对瘢痕疙瘩的厚度、大小进行客观的评价。我们在此基础上，采用彩色多普勒对瘢痕疙瘩的血供情况加以评分，剪切波弹性成像间接评估瘢痕疙瘩的硬度，更有利于对瘢痕疙瘩的严重程度、活跃程度进行评价，与临床关系密切。

瘢痕疙瘩的治疗方法包括手术切除，放射线照射，糖皮质激素注射，冷冻治疗，硅酮类药物及凝胶等。但目前为止，尚没有哪种方法能获得满意的治愈率。临床治疗以多种方法的联用为主，例如手术结合放疗、手术结合注射等。对瘢痕疙瘩患者采用注射治疗也是一种常用的方法，据上海第九人民医院武小莉报道，将糖皮质激素结合微量 5-FU 进行病变内注射，可以有效的闭锁瘢痕疙瘩的血管，缩小瘢痕疙瘩体积，继而通过长期的逐渐减量的注射过程达到彻底根治的目的。但激素并发症如 Cushing 综合征、痤疮等的发生，注射时的剧烈疼痛，长期多次的注射，均让很多患者放弃治疗，瘢痕疙瘩重新复发。据文献报道，手术结合放射线照射的复发率也存在较大的差异性，从 20%-80%不等，原因可能与放疗方法不同，纳入统计的瘢痕疙瘩发生部位不一致，对复发的衡量标准不一样有关。不同部位的瘢痕疙瘩复发几率存在很大差异，据 Ogawa 报道，经过手术结合放射线照射的综合治疗，耳垂的复发几率约 5%，胸肩背部约 30%，身体其他部位的复发率介于两者之间。放射线照射的具体方法也一直存在争议。接触性放疗和直线加速器的离子照射是最常用的方法，照射方案包括大剂量单次照射，小剂量多次照射等。

本课题研究者采用将药物注射在瘢痕疙瘩和皮下的交界层结合瘢痕疙瘩内注射的方式，希望获得更好的临床疗效。经过 2-3 次注射，确保瘢痕疙瘩与皮肤平齐后加以 Sr90 的接触性放射治疗。本课题旨在对该方法的临床效果、副作用进行观察和评价。

2 研究目的

本课题研究者采用瘢痕疙瘩内注射和基底注射相结合的方式，希望获得更好的临床疗效，开发出能减轻患者注射时的痛苦，提高依从性，增加临床效果的注射方法。经过注射治

疗，确保瘢痕疙瘩与皮肤平齐后加以 Sr90 核素敷贴的接触性放射治疗。通过与对照组的比较，观察瘢痕疙瘩的治疗效果和复发情况，旨在评价该治疗的临床效果，了解副作用的发生情况，希望能提高临床治愈率，减少瘢痕疙瘩的复发。

3 研究方法

3.1 第一部分

依据主要终点事件随访终点的瘢痕疙瘩厚度（彩超测量）理想预测值估算样本量，根据计量资料优劣性临床试验的样本量计算公式计算样本量：

$$n = 2\sigma^2 \times f(\alpha,\beta)/(\mu_1-\mu_2)^2$$

依据前期研究基础，对照组均值 μ_1=3.05，标准差σ=0.69；设定主要疗效指数临床有意义的差值$\mu_1-\mu_2$为 0.60；α=0.05，power=80%。考虑可能有 10%失访率，扩大样本量，最终设定研究样本为每组 40 例，总例数为 80 例。

3.1.1 纳入标准：18 岁以上确诊瘢痕疙瘩的患者，病程超过 2 年且没有自发萎缩倾向。患者同意治疗和检测并签署知情同意书。排除标准：患者的瘢痕疙瘩在 3 个月内接受过手术、放疗、注射、冷冻、激光等治疗。患者不同意使用 TAC+5-Fu 的药物注射方案。瘢痕疙瘩伴感染或囊肿的病例。瘢痕疙瘩伴有其他皮肤病，如皮疹。大小超过 9 cm^2，厚度超过 4mm 的瘢痕疙瘩。耳垂瘢痕疙瘩。伴随其他严重系统疾病患者，如糖尿病、免疫性疾病、恶性肿瘤、心血管疾病、呼吸系统疾病等。

3.1.2 采集患者的性别、年龄、评估瘢痕疙瘩的 VSS，彩色多普勒检测瘢痕疙瘩；

3.1.3 将纳入患者随机分为瘢痕疙瘩病变内注射组和瘢痕疙瘩基底结合病变内注射组，于患者开始注射前、注射后 1、2、3 月评估 VSS；于开始注射前、注射后第 3 月使用彩色多普勒评估瘢痕疙瘩厚度、硬度、血液流速；每次注射治疗后评估患者疼痛程度；激素注射副作用及复发情况随访至开始治疗后 1 年。

3.2 第二部分

4.1 临床试验样本量：

依据主要终点事件随访终点的瘢痕疙瘩厚度（彩超测量）理想预测值估算样本量，根据计量资料优劣性临床试验的样本量计算公式计算样本量：

$$n = 2\sigma^2 \times f(\alpha,\beta)/(\mu_1-\mu_2)^2$$

依据前期研究基础，设定主要疗效指数临床有意义的差值$\mu_1-\mu_2$为 0.83；α=0.05，power=80%。考虑可能有 10%失访率，扩大样本量，最终设定研究样本为每组为 21 例，

总例数为42例。

3.2.1 纳入标准：18岁以上确诊瘢痕疙瘩的患者，病程超过2年且没有自发萎缩倾向。患者同意治疗和检测并签署知情同意书。已经接受注射治疗，且患者同意继续接受放射线照射。排除标准：患者的瘢痕疙瘩在3个月内接受过手术、放疗、注射、冷冻、激光等治疗。患者不同意使用TAC+5-Fu的药物注射方案。瘢痕疙瘩伴感染或囊肿的病例。瘢痕疙瘩伴有其他皮肤病，如皮疹。大小超过9 cm^2，厚度超过4mm的瘢痕疙瘩。耳垂瘢痕疙瘩。伴随其他严重系统疾病患者，如糖尿病、免疫性疾病、恶性肿瘤、心血管疾病、呼吸系统疾病等。

3.2.2 采集患者的性别、年龄、评估瘢痕疙瘩的VSS，彩色多普勒检测瘢痕疙瘩；

3.2.3 将42例注射后瘢痕疙瘩随机分为实验组和对照组。实验组接受总剂量为15-20Gy的90Sr同位素放疗，对照组在注射后不再接受放疗。

3.2.4 辅助放疗组患者于放疗前、放疗完成后每个月进行VSS评估；于放疗前、放疗完成后第3月、6月、1年使用彩色多普勒评估瘢痕疙瘩厚度、硬度、血液流速；单纯注射治疗组患者于同期完成各项评估。如果患者的VSS评分增加1，则视为复发，我们将停止随访并即刻做彩超检测作为终点指标。随访期间出现的并发症如色素沉着、月经紊乱、局部凹陷、皮肤溃疡、Cushing综合征等均作如实记录。

4 **研究步骤**

第一部分

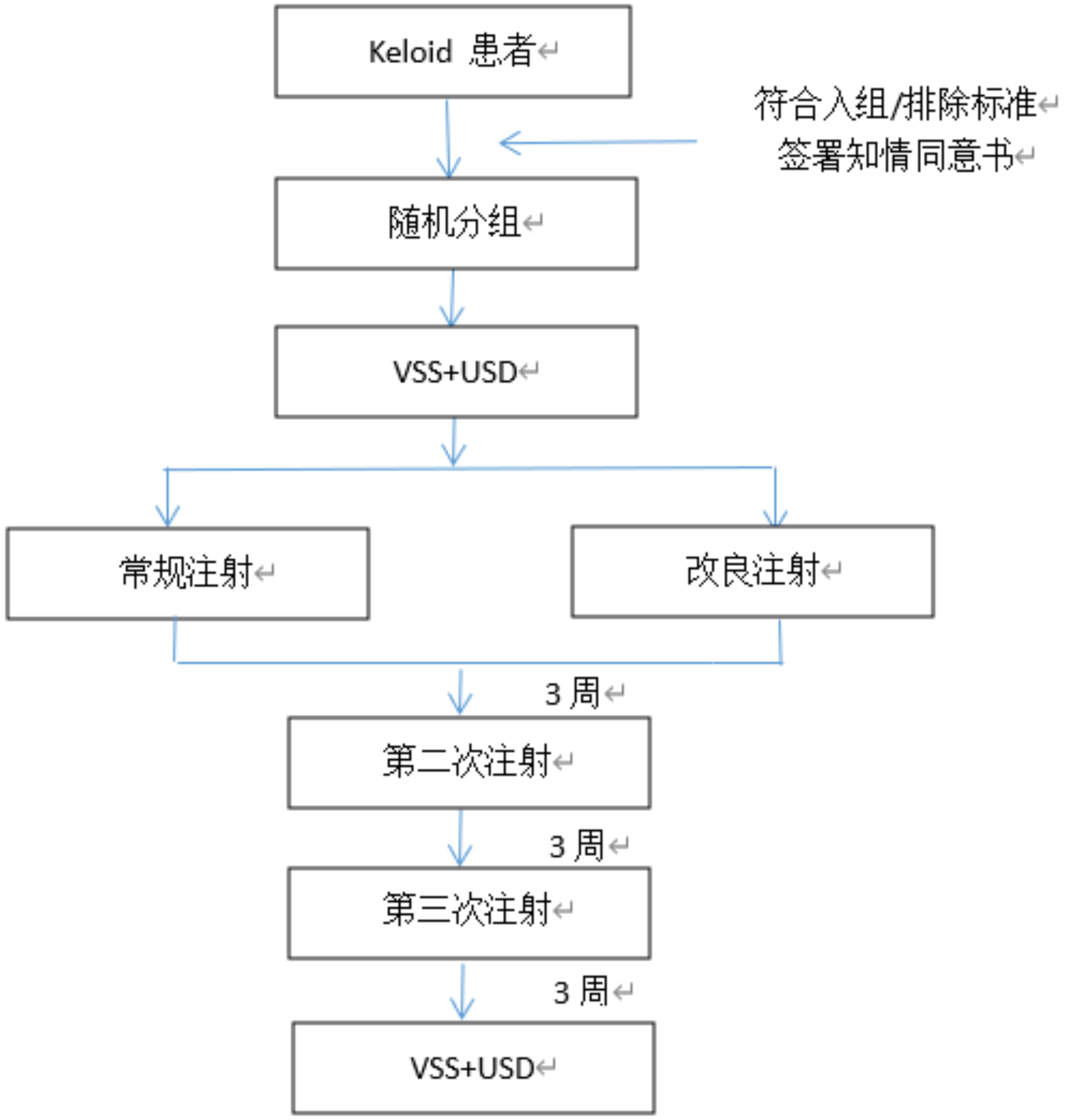
Keloid 患者
符合入组/排除标准
签署知情同意书
随机分组
VSS+USD
常规注射
改良注射
3 周
第二次注射
3 周
第三次注射
3 周
VSS+USD

第二部分

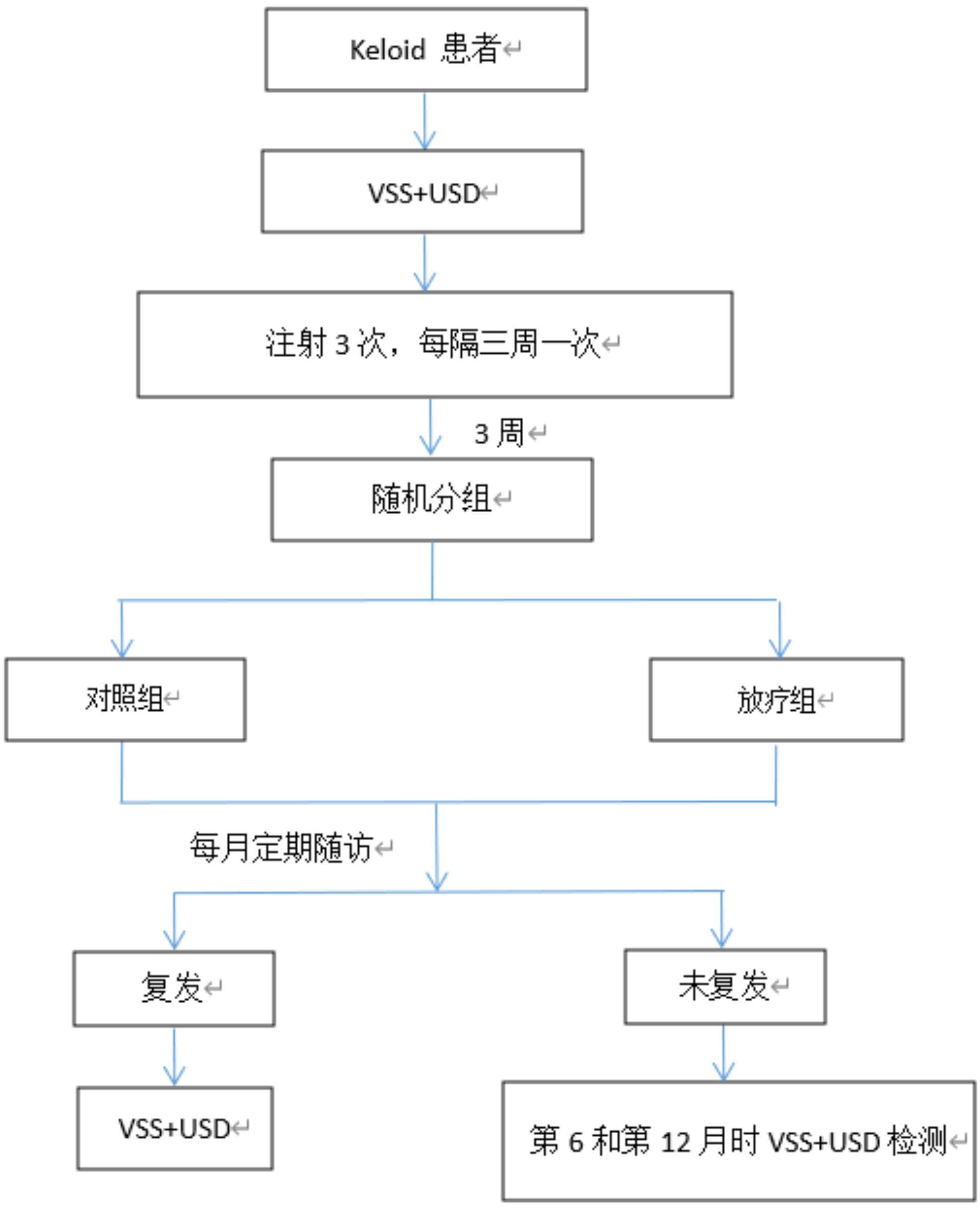

5 可供选择的其他治疗方法

如患者不愿意接受注射治疗，可以采用手术结合放射线照射或单纯瘢痕贴外用的治疗方案。

6 风险与收益

风险分析：注射过程中患者可能感紧张、疼痛，可能出现头晕、心悸、面色苍白、冷汗、恶心欲吐等晕针症状；注射后存在发生月经紊乱、痤疮、Cushing 综合征的可能。放射线照射后可能发生皮炎、皮肤破溃、色素沉着、潜在的致癌风险。至少治疗结束后半年

才能怀孕或哺乳。受益分析：这一研究工作的完成有希望改进传统的瘢痕疙瘩注射治疗的方法，减轻患者疼痛，提高依从性，增进临床治疗效果。注射治疗结合核素照射的治疗方式有希望提高临床治愈率，减少瘢痕疙瘩的复发机会。

7 不良事件观察、记录和处置

7.1 不良事件分类：

轻度不良事件：月经紊乱、局部个别痤疮新发等；

中度不良事件：皮下组织轻度凹陷、局部多发痤疮

严重不良事件：皮肤溃疡、皮下组织明显凹陷、全身多发痤疮、严重的药物过敏性休克

7.2 研究中的风险预防和应对措施：

研究者及时判断和确定不良事件的程度，并对不良事件进行以下分级处理：轻度不良事件，继续观察；中度不良事件，适当减少药量，到皮肤科应用维甲酸、美满霉素等药物治疗痤疮；严重不良事件，应立即停止并通知研究负责人，并上报上级部门，组织相关专家进行会诊，制定有效的治疗和抢救方案。皮肤溃疡应积极采用促进创面愈合的药物或敷料，避免创面感染或加重；皮下组织明显凹陷停药后以观察为主；痤疮明显加重患者到皮肤科应用维甲酸、美满霉素等药物治疗。严重药物过敏性休克需要立即启动抢救预案。

7.3 研究者及时完成不良事件和严重不良事件的记录，内容包括：不良事件和严重不良事件的详细描述；不良事件和严重不良事件的发生时间、持续时间、处理方案；如事件需要用药物治疗的，记录治疗药物的途径、剂量、治疗时间和理由等，并记录治疗结果。

7.4. 对严重不良事件在 24 小时内向医院伦理委员会报告。

8 研究评估

严格按照 GCP 对临床试验过程进行监查和稽查，确保临床试验数据真实、可信。坚决杜绝研究过程中追求经济利益而弄虚作假。

9 研究质量控制和保证

9.1 在实验开始前，由研究负责人对全体研究人员进行培训，培训内容包括：相关法规、本临床实验的各项技术，VSS 瘢痕评价系统以及管理制度。

9.2 研究负责人在实验开始后，随时检查相关研究人员的具体操作及各项原始资料的记录是否及时、准确、规范，一旦发现问题，及时改正。

9.3 接受医院临床试验办公室的监督和检查，并对其提出的问题及时改正。

10 伦理学要求

必须遵循《中华人民共和国药品管理法》、《药物临床试验质量管理规范》、《医疗器械临床试验规定》、《世界医学大会赫尔辛基宣言》、《涉及人的生物医学研究伦理审查办法(试行)》等伦理准则，以及世界卫生组织有关伦理审查等指南，从保障受试者权益与安全的角度，严格审查临床试验研究方案。在研究开始之前，由医学研究负责单位的伦理专委会批准该试验方案后方可实施临床试验。每一位病人入选本研究前，研究医师有责任以书面文字形式，向其或其指定代表完整、全面地介绍本研究的目的、程序和可能的风险。应让患者知道他们有权随时退出本研究。人选前须给每位患者一份书面患者知情同意书（以附录形式包括于方案中），研究医师有责任在每位患者进人研究之前获得知情同意。知情同意书应作为临床研究文档保留备查。

11 组长单位主要研究者签名和日期

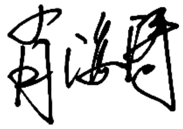

12 课题组参加人员

<table>
<tr><td rowspan="10">项目人员（研究人员简历见申请书附件）</td><td colspan="4">项目负责人</td></tr>
<tr><td>姓名</td><td>肖海涛</td><td>所在科室</td><td>美容整形烧伤外科</td></tr>
<tr><td>职务/职称</td><td>主治医师</td><td>研究方向</td><td>病理性瘢痕基础与防治</td></tr>
<tr><td>联系电话</td><td>18980606392</td><td>邮箱</td><td>xhaitao@163.com</td></tr>
<tr><td colspan="4">研究人员名单及分工</td></tr>
<tr><td>姓名</td><td>学位/职称</td><td>专业</td><td>主要任务</td></tr>
<tr><td>邓珂</td><td>博士在读</td><td>整形美容烧伤</td><td>资料记录、患者随访</td></tr>
<tr><td></td><td></td><td></td><td></td></tr>
</table>
