# Supplementary material for: Strontium-90 brachytherapy following intralesional triamcinolone and 5-fluorouracil injections for keloid treatment: A randomized controlled trial
Source: PLoS One. 2021 Mar 23;16(3):e0248799. doi: 10.1371/journal.pone.0248799 (PMC7987169; doi:10.1371/journal.pone.0248799)
Supplement: S3 File — (PDF) [file pone.0248799.s004.pdf]

Biomedical Ethics Research Program (Interventional clinical research)

"Evaluation of the clinical efficacy of Modified injection technique and combined Sr90 isotope irradiation in the treatment of keloids" Study Protocol

Hospital: West China Hospital

Applicant: Haitao Xiao

Department: Department of burn and plastic surgery

Telephone: 18980606392

Date: Jan. 2019-July. 2020

Version：V2.1

Abstract of the Protocol

**Research Design**	**□Case-control study □Cohort study □Cross-sectional study**<br>**√Randomized controlled study □Blind method □Others**
**Research type**	**（A：High risk）**<br>□ Gene editing research<br>□ Cell therapy research<br>□ Implantable medical device research<br>□ Class III new clinical technology (safety and effectiveness, high technical difficulty, high risk)<br>□ Special population research (children, pregnant women, people with mental retardation, etc.)<br>□ Off-label drug use<br>□ off-specification equipment use<br>□ Others<br>**（B：Middle risk）**<br>□ Post-marketing biologics research(Prevention and treatment use)<br>□ Post-marketing therapeutic vaccine research<br>□ Post-marketing rare disease drug research<br>√ Class Ⅱ new clinical technology (safe and effective, certain technical difficulty, certain medical risk and ethical risk)<br>□ Others<br>**（C：Low risk）**<br>□ 5 years of drug research on the market (including chemical drugs, generic drugs, etc.)<br>□ Research on listed devices (including AI, imaging software)<br>□ Class I new clinical technology (medical technology with precise safety and effectiveness, low technical difficulty, and almost no ethical risk)<br>□ Others

**Total number of cases**	122
**Risk/benefit analysis**	The modified injection technique can reduce the pain during injection and speed up the atrophy of keloids. However, compared to traditional injection methods, the new technique injects closer to the base of the keloid, which may increase the risk of tissue depression. Adding adjuvant radiotherapy after injection is expected to reduce the recurrence rate of keloid patients, how ever, low-dose radiotherapy may increase the risk of pigmentation.
**Risk judgment**	□Not more than the minimum risk √Greater than minimum risk<br>Minimal risk: refers to the probability and degree of the expected risk in the experiment which is not greater than the risk of daily life, or routine physical examination or psychological test
**Research period**	Jan. 2019-July. 2020

1 Background

Keloid is a disease caused by abnormal proliferation of fibroblasts and accumulation of extracellular matrix. The clinical manifestations are hard protruding scars on the skin, often accompanied by itching and pain. The main difference from ordinary scars is that the size of the lesion exceeds the scope of the original injury, and it grows in a crab-like shape to the surrounding area without shrinking by itself.

In the past, keloids were mainly evaluated by the Vancouver Scar scale(VSS). The Vancouver Scar Scale mainly judges the thickness, hardness, color, and blood flow of keloids by doctors' face-to-face consultation, and makes subjective scores accordingly. However, the evaluation of different doctors varies greatly. Later, some people introduced color Doppler ultrasound, three-dimensional CT and other methods to objectively evaluate the characters of keloids. Based on the reports, we use color Doppler to score the blood supply of keloids, and shear wave elastography to evaluate the hardness of keloids.

The treatment of keloids includes surgical resection, radiation therapy, glucocorticoid injection, cryotherapy, silicone gels, etc. So far, no method alone can get a satisfactory cure rate. Clinical treatment is based on a combination of multiple methods, such as surgery combined with radiotherapy, surgery combined with injection, etc. Injection therapy for keloid patients is a first-line treatment. According to Wu, intralesional injection of glucocorticoid combined with small dose of 5-FU can effectively block the blood vessels and reduce the volume of the keloid. However, the complications of injection include Cushing syndrome, acne, severe pain during injection. Repeated injections over a long period of time have caused many patients to give up. According to the literature, the recurrence rate of surgery combined with radiation exposure varies greatly from 20% to 80%. According to articles by Ogawa, after a comprehensive treatment of surgery and radiation irradiation, the recurrence rate of the earlobe is about 5%, the chest, shoulder and back is about 30%, and the recurrence rate of other parts of the body is between the two. between. The specific method of radiation exposure has also been controversial. Brachytherapy of radiation and electron beam irradiation are the commonly used methods. The irradiation plan includes a single large-dose irradiation and multiple small-dose irradiations.

Researchers in this subject adopt the method of injecting drugs firstly into keloid's base and then injecting into the parenchyma, hoping to obtain a better clinical effect. After 2-3 injections

which ensure the flatness of keloid, Sr90 brachytherapy is applied. This research aims to observe and evaluate the clinical effects and side effects of this method.

2 Purpose

Researchers use a combination of basal and intralesional injection, hoping to obtain better clinical efficacy while reducing the pain. After injection, brachytherapy with Sr90 isotope is applied. By comparing with the control group, the effects, complications and recurrence rate were observed, in order to evaluate the clinical effect of the treatment.

3 Research method

3.1 Part 1:

3.1.1 Based on an estimated effect size of d = .60, alpha level = .05 (one-tailed as direction was hypothesised), power = .80, we estimated that we would require a total of n = 36 lesions per group and therefore aimed to recruit 40 lesions per group based on a 10% dropout rate.

3.1.2 Inclusion criteria: (1) Patients who were over 18 years old met the clinical diagnostic criteria of keloids: scar-like structures resulted from wound healing, protruding skin surface, infiltrating beyond original damage area. It might be accompanied by itching, pain and other discomfort symptoms. (2) The disease course was longer than 2 years and no spontaneously shrinking tendency was noticed. (3) Patients signed the informed consent and agreed to complete the follow-up procedure including VSS (Vancouver Scar Scale) assessment and color Doppler ultrasound assessment at the designated visit time.

Exclusion criteria: (1) Patients who were previously treated with surgery, radiation, injection, laser, cryotherapy, etc. within 3 months. (2) Patients who were unable to complete VSS evaluation and color Doppler ultrasound evaluation; (3) Patients refused to accept ingredient including TAC or 5-Fu; (4) Keloids experienced infection or ulceration. (5) Other skin conditions including rash were involved; (6) Patients with systemic diseases such as hypertension, diabetes, gout, or immune diseases; (7) Patients who were allergic to injection therapy; (8) Patients with ear lobe keloids; (9) Patients with keloids larger than 9 cm^2 or thicker than 4mm.

3.1.3 Collect the patient's information, including gender, age, VSS, and color Doppler results;

3.1.4 Included patients were allocated equally to either the experimental group or the control group using the block randomization method. The conventional injection technique was performed in the

control group, while the modified injection technique was performed in the experimental group.

3.1.5 The VSS was evaluated before the injection, 1, 2, and 3 months after the injection; Color Doppler was used to evaluate the thickness, hardness, and blood flow rate of the keloid before the injection and the third month after the injection; The pain degree was evaluated after each injection; side effects and recurrence are followed up to 1 year.

3.2 Part 2

3.2.1 Based on an estimated effect size of d = .83, alpha level = .05 (one-tailed as direction was hypothesised), power = .80, we estimated that we would require a total of n =19 lesions per group and therefore aimed to recruit 21 lesions per group based on a 10% dropout rate.

3.2.2 Inclusion criteria: (1) Patients who were over 18 years old met the clinical diagnostic criteria of keloids: scar-like structures resulted from wound healing, protruding skin surface, infiltrating beyond original damage area. It might be accompanied by itching, pain and other discomfort symptoms. (2) The disease course was longer than 2 years and no spontaneously shrinking tendency was noticed. (3) The patients have already received injection therapy, and agrees to continue to receive radiation exposure (4) Patients signed the informed consent and agreed to complete the follow-up procedure including VSS (Vancouver Scar Scale) assessment and color Doppler ultrasound assessment at the designated visit time.

Exclusion criteria: (1) Patients who were previously treated with surgery, radiation, injection, laser, cryotherapy, etc. within 3 months. (2) Patients who were unable to complete VSS evaluation and color Doppler ultrasound evaluation; (3) Patients refused to accept ingredient including TAC or 5-Fu; (4) Keloids experienced infection or ulceration. (5) Other skin conditions including rash were involved; (6) Patients with systemic diseases such as hypertension, diabetes, gout, or immune diseases; (7) Patients who were allergic to injection therapy; (8) Patients with ear lobe keloids; (9) Patients with keloids larger than 9 cm^2 or thicker than 4mm.

3.2.3 Collect the patients' information, including gender, age, VSS, and color Doppler results;

3.2.4 Included patients after injection were randomly divided into experimental group and control group. The experimental group received 90Sr isotope radiotherapy with a total dose of 15-20Gy, and the control group no longer received radiotherapy after injection. VSS scoring was performed 3 weeks after the last injection and monthly after the brachytherapy completion or any time when

patients visited for recurrence concern. Color Doppler ultrasound examination was performed 3 weeks after the last injection and 6、12 months after the brachytherapy completion. Patients' subjective symptoms including itching and pain were recorded. If a patient's VSS thickness score increased by 1 after the whole treatment course was completed, the follow-up would be ended and we would perform an VSS assessment and color Doppler ultrasound examination as an endpoint evaluation before re-injection. Complications such as hyperpigmentation, menstrual disorders, local depression, skin ulceration, and Cushing syndrome were recorded during the follow-up.

4 Protocol

Part 1:

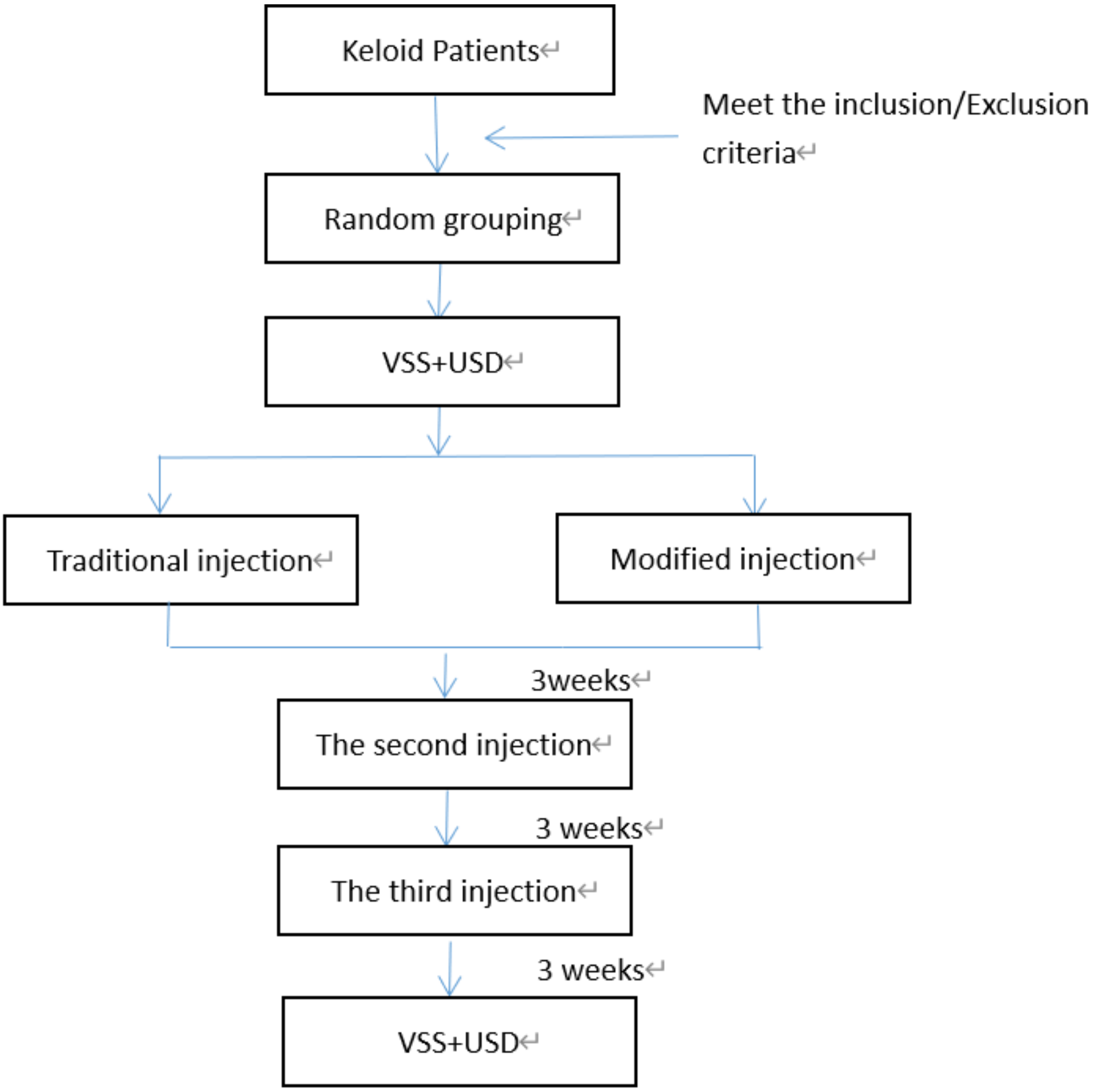

Part 2:

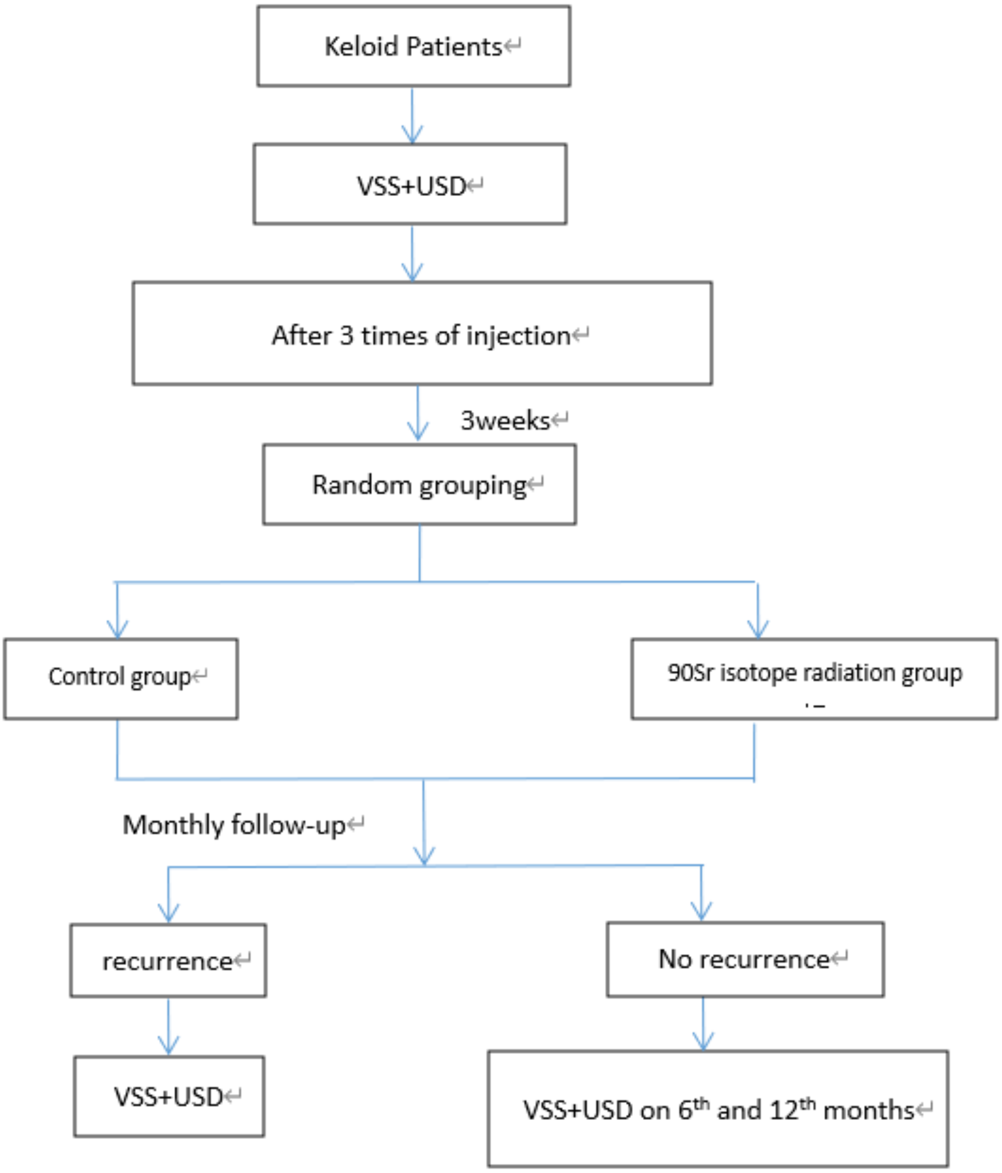

5 Other treatment available

If the patient is unwilling to receive injection therapy, a treatment plan that combines surgery with radiation irradiation or simple scar sticking for external use can be used.

6 Risks and benefits

Risk analysis: During the injection, patients may feel nervous and painful, and may experience dizziness, palpitations, pale complexion, cold sweats, nausea and vomiting; menstrual disorders, acne, and Cushing syndrome may occur after injection. Dermatitis, skin ulceration, pigmentation, and potential carcinogenic risks may occur after radiation exposure. At least half a year after the

end of treatment can the patient be suggested pregnant or breastfeeding

Benefit analysis: The completion of this research work may improve the traditional keloid injection treatment method, reduce patient pain, improve compliance, and improve clinical treatment effects. The combination of injection therapy and brachytherapy is expected to increase the clinical cure rate and reduce the chance of recurrence of keloids.

7. The observation, recording and handling of adverse events

7.1 Classification of adverse events:

Mild adverse events: menstrual disorders, new local acne, etc.;

Moderate adverse events: mild depression of subcutaneous tissue, local multiple acne

Serious adverse events: skin ulcers, obvious depression of subcutaneous tissues, multiple acne throughout the body, severe drug-induced anaphylactic shock

7.2 Risk prevention and response measures in research

The investigator judges and determines the degree of adverse events, and handles the adverse events accordingly. Mild adverse events, continue to observe; Moderate adverse events, reduce the dosage used, if acne occurs, use retinoic acid, minocycline or other drugs; Serious adverse events, immediately stop treatment and notify the person in charge of the study, report to the superior department, organize relevant experts to conduct consultations, and formulate effective treatment. Skin ulcers can be treated with drugs that promote wound healing; patients with obviously aggravated acne should be treated with retinoic acid and minocycline in the dermatology department. If severe drug anaphylactic shock happens, rescue process should be started immediately

7.3 The investigator should complete the records of adverse events and serious adverse events in time, including: detailed descriptions of adverse events and serious adverse events; the occurrence time, duration, and treatment plan of adverse events and serious adverse events; If the event requires medication, record the route, dosage, time and reason, the result should also be recorded.

7.4 Report serious adverse events to the hospital ethics committee within 24 hours.

8 Evaluation

In strict accordance with the GCP, the clinical trial process is monitored and audited to ensure the authenticity and credibility of the clinical trial data. Resolutely put an end to fraud in the pursuit of economic interests in the research.

9 Quality control

9.1 Before the start of the experiment, the research leader will train all the researchers. The content includes: relevant regulations, various techniques and management systems of this clinical experiment， Vancouver scar evaluating system.

9.2 After the start of the experiment, the person in charge of the research will check whether the specific operations of the relevant researchers and the records of the original data are timely, accurate, and standardized. Once problems are found, they should be corrected in time.

9.3 Accept the supervision and inspection of the clinical trial office of the hospital, and correct the problems in time.

10 Ethical requirements

Must comply with the "Pharmaceutical Administration Law of the People's Republic of China", "Pharmaceutical Clinical Trial Quality Management Regulations", "Medical Device Clinical Trial Regulations", "World Medical Congress Helsinki Declaration", "Measures for Ethical Review of Biomedical Research Involving People (Trial)" Such ethical guidelines, as well as the World Health Organization’s guidelines for ethical reviews, strictly review clinical trial research programs from the perspective of protecting the rights and safety of patients. Before the start of the study, the clinical trial can only be implemented after the ethics committee of the medical research unit approves the trial protocol. Before each patient is selected for this study, it is the responsibility of the physician to fully and comprehensively introduce the purpose, procedures and possible risks of this study to him. Patients should be aware that they have the right to withdraw from the study at any time. A written informed consent form (included in the protocol in the form of an appendix) must be given to each patient before candidates are selected, and the physician is responsible for obtaining informed consent before each patient enters the study. The informed consent form should be kept as a clinical research document for future reference.

11 Signature and date of investigators

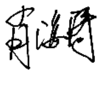

12 Research participants

Personnel information of the	Project manager			
	Name	Haitao Xiao	Department	Burn and Plastic Surgery

research	Position	Physicist	Research direction	The etiology and treatment of pathological scar
	Telephone	18980606392	E-mail	xhaitao@163.com
	Researchers			
	Name	Ke Deng	Department	Burn and Plastic Surgery
	Position	Physicist	Main task	Data records, patient follow-up
